# Intrarenal venous flow patterns and their association with successful fluid removal in critically ill patients: a prospective observational exploratory study

**DOI:** 10.1186/s13089-025-00447-z

**Published:** 2025-10-06

**Authors:** Chailat Maluangnon, Apatsara Saokaew, Satit Rojwatcharapibarn, Ranistha Ratanarat

**Affiliations:** 1 Division of Critical Care, Department of Medicine, Faculty of Medicine Siriraj Hospital, Mahidol University, Bangkok, Thailand; 2 Department of Radiology, Faculty of Medicine Siriraj Hospital, Mahidol University, Bangkok, Thailand

**Keywords:** De-escalation, Doppler ultrasound, Fluid removal, Intensive care unit, Intrarenal venous flow, Venous excess ultrasound

## Abstract

**Background:**

Determining the optimal timing for fluid removal in critically ill patients remains a challenge. This study evaluated the utility of Doppler ultrasound, specifically intrarenal venous flow (IRVF) patterns and venous excess ultrasound (VExUS) scores, and their associations with fluid removal outcomes, hemodynamic parameters, and clinical endpoints.

**Methods:**

In this prospective observational exploratory study, 52 intensive care unit (ICU) patients who underwent fluid removal were enrolled. Baseline IRVF patterns and VExUS scores were assessed, with follow-up evaluations performed daily for three days. The primary outcome was to evaluate whether IRVF patterns were associated with successful fluid removal, defined as achieving a negative fluid balance for at least two consecutive days. Secondary outcomes included associations with central venous pressure (CVP), NT-proBNP, cumulative fluid balance, and clinical outcomes.

**Results:**

Thirty-one patients (59.6%) achieved successful fluid removal. A discontinuous baseline IRVF pattern was independently associated with successful fluid removal (adjusted odds ratio 4.31, 95% CI 1.02–18.18; P = 0.047). This pattern demonstrated high sensitivity of 87.1% (95% CI 70.2–96.4), moderate specificity of 42.9% (95% CI 21.8–66.0), and accuracy of 69.2% (95% CI 54.9–81.3). VExUS scores grades 2–3 demonstrated high specificity of 85.7% (95% CI 63.7–97.0) but low sensitivity of 29.0% (95% CI 14.2–48.0), with an accuracy of 51.9% (95% CI 37.6–66.0). An improvement in the IRVF pattern was significantly correlated with a reduction in NT-proBNP levels (P = 0.048). However, neither IRVF patterns nor VExUS scores improvements were associated with changes in fluid balance, CVP, or clinical outcomes such as 28-day mortality, ventilator-free days, or ICU length of stay.

**Conclusions:**

Discontinuous IRVF patterns at baseline were significantly associated with fluid removal success, representing a physiologically based marker for deresuscitation readiness. More large-scale studies are warranted to validate these findings and explore long-term implications.

*Trial registration* ClinicalTrials.gov identifier NCT06216119. Registered 22 January 2024, https://clinicaltrials.gov/study/NCT06216119

**Supplementary Information:**

The online version contains supplementary material available at 10.1186/s13089-025-00447-z.

## Introduction

### Background

Effective fluid resuscitation is crucial for stabilizing tissue perfusion in critically ill patients with shock. Fluid therapy follows four distinct phases: Rescue, Optimization, Stabilization, and De-escalation (ROS-D) [1]. While early resuscitation is vital, excessive fluid accumulation can lead to venous congestion, organ dysfunction, and adverse clinical outcomes, including increased mortality, acute kidney injury (AKI), prolonged intensive care unit (ICU) stays, and extended duration of mechanical ventilation [2–5]. The de-escalation phase aims to achieve a negative fluid balance while preventing hypotension and hypoperfusion [3, 6]. However, determining the optimal time for fluid removal remains challenging due to the lack of precise and reliable bedside assessment tools.

Traditional methods to assess volume status, including clinical examination, imaging, physiological parameters, biomarkers, and bioimpedance analysis, have limitations in detecting early venous congestion [4, 5]. Point-of-care ultrasound (POCUS) has emerged as a valuable bedside tool for real-time, noninvasive volume assessment and enables readily performed follow-up scans. The Venous Excess Ultrasound (VExUS) score quantifies systemic congestion by integrating 2D and Doppler imaging to assess the inferior vena cava (IVC), hepatic, portal, and intrarenal veins [7, 8].

Intrarenal venous flow (IRVF) assessment provides a direct evaluation of renal venous congestion, offering an alternative approach to the assessment of fluid status. The kidneys are particularly sensitive to venous congestion, as elevated venous pressure can cause backpressure in encapsulated organs [5]. In heart failure patients, discontinuous IRVF patterns have been associated with worsening congestion and adverse clinical outcomes [9, 10]. Furthermore, studies suggest that IRVF patterns fluctuate with congestion levels, with patients exhibiting discontinuous renal venous flow during congestive states, which normalizes to a continuous pattern following decongestion [9, 11]. However, these findings may not be fully applicable to septic ICU patients, where IRVF patterns were not correlated with central venous pressure (CVP) but were associated with acute kidney injury (AKI) and mortality [12]​. This suggests that IRVF patterns could serve as an indicator of venous congestion and may function as a bedside tool to predict clinical outcomes. Despite these potential implications, the role of IRVF in critically ill ICU patients undergoing fluid removal remains uncertain, warranting further investigation.

Given this knowledge gap, the assessment of IRVF may serve not only as a marker of congestion, but also as a potential guide for fluid removal strategies and monitoring responses to decongestive therapy. However, no studies have comprehensively examined the relationship between IRVF patterns, VExUS scores, and clinical outcomes in critically ill patients undergoing fluid removal.

Therefore, this study aims to evaluate the utility of Doppler ultrasound—specifically IRVF patterns and VExUS scores—as tools associated with fluid removal success and to assess its correlation with hemodynamic parameters and clinical outcomes.

## Methods

### Study design and setting

This was a prospective exploratory observational study conducted at Siriraj Hospital, a tertiary university hospital in Bangkok, Thailand. Prior to the start of the study and enrollment of the first patient, the protocol was approved by the Siriraj Institutional Review Board (Si929/2023) and prospectively registered with ClinicalTrials.gov (NCT06216119). The study was conducted in accordance with the ethical principles outlined in the Declaration of Helsinki of the World Medical Association. Informed consent was obtained from each patient or their legally authorized representative before enrollment.

### Participants

We screened ICU patients from January 23, 2024, to September 13, 2024. Eligible patients were adults (≥ 18 years) admitted to the medical ICU with an expected stay in the ICU of more than 120 h. The patients were required to have hemodynamic stability without the need for high-dose vasoactive support. Fluid removal was initiated at the discretion of the attending physician using diuretics or renal replacement therapy (RRT).

Patients were excluded if they had chronic kidney disease (eGFR < 30 mL/min/1.73 m^2^), preexisting RRT, decompensated cirrhosis with portal hypertension, venous thrombosis (IVC, portal, hepatic, or renal veins), ureteral obstruction, intra-abdominal hypertension (> 12 mmHg), a history of diuretic allergy, pregnancy, prior kidney or liver transplantation, or do-not-resuscitate (DNR) orders. Patients who were unable to provide informed consent or whose legally authorized representatives declined participation were excluded.

Patients were withdrawn from the study if they developed a new-onset shock that required rapid fluid resuscitation within 72 h of enrollment. Withdrawal could also occur at the discretion of the patient, their family, or the attending physician (Additional File1).

### Outcomes

The primary outcome was to evaluate whether IRVF patterns were associated with successful fluid removal, defined as achieving a negative fluid balance for at least two consecutive days without predefined treatment-limiting adverse events (e.g., new-onset shock). Secondary outcomes included correlations between improvements in IRVF patterns (a transition from discontinuous to continuous pattern from baseline to day 3) and reductions in VExUS scores (from baseline to day 3) with CVP, NT-proBNP levels, cumulative fluid balance and clinical outcomes, including 28-day mortality, ICU length of stay, hospital length of stay, RRT-free days, and ventilator-free days.

### Ultrasonography assessments

**Fig. 1:**
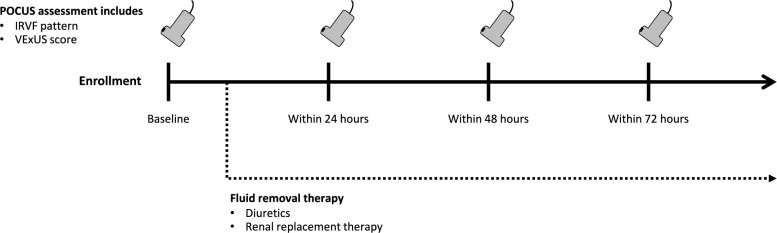
Study protocol. IRVF, Intrarenal venous flow; VExUS, Venous Excess Ultrasound

After enrollment, patients underwent baseline ultrasonography to assess IRVF patterns, VExUS scores, and cardiac function before initiating fluid removal therapy with diuretics or RRT. Serial ultrasonography assessments were conducted within 24, 48, and 72 h, along with data collection (Fig. 1).

All ultrasound examinations were performed by a single investigator trained in POCUS. To ensure accuracy, the investigator completed specialized training in renal and VExUS Doppler assessments and performed ten supervised examinations in the ICU prior to the study. Doppler waveforms, recorded with concurrent electrocardiogram (ECG) traces, were independently reviewed by a blinded intensivist and a radiologist to minimize bias. Both interpreters were blinded to patient identity, clinical data, and outcomes. In cases of disagreement, the final interpretation was determined through consensus with a senior intensivist. Patients with inadequate ultrasound images were excluded from waveform analysis. Treating physicians remained blinded to all ultrasound results to maintain objectivity.

### Intrarenal venous flow (IRVF) pattern assessment

Renal Doppler ultrasonography was performed using a commercially available system equipped with a convex transducer (frequency range: 2–6 MHz). The right kidney was examined in the coronal plane with the patient in the supine position, except in cases where postural restrictions were applied. Color Doppler imaging was used to identify interlobar vessels, and pulsed Doppler waveforms of interlobar arteries and veins were recorded simultaneously. To enhance timing accuracy and improve waveform interpretation, all Doppler acquisitions were performed with concurrent ECG tracing.

**Fig. 2:**
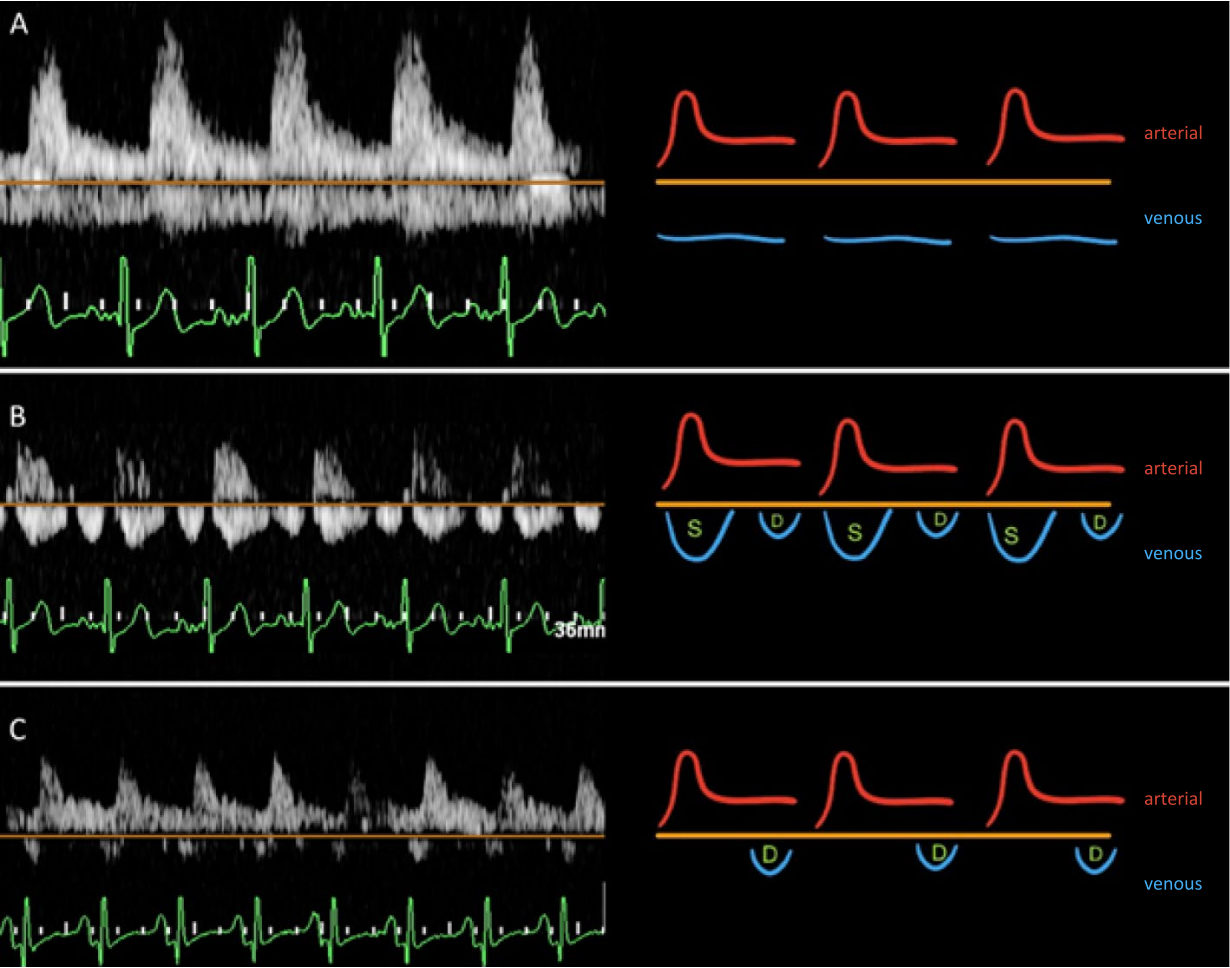
Intrarenal flow pattern. **A** Continuous flow pattern; **B** biphasic discontinuous pattern; **C** monophasic discontinuous pattern

IRVF patterns, characterized by flow away from the transducer below the baseline, were categorized into two main types based on the classification by Iida et al. [10]: continuous and discontinuous patterns. Discontinuous patterns were further subclassified into biphasic discontinuous and monophasic discontinuous patterns (Fig. 2).

### Venous excess ultrasound (VExUS) score assessments

VExUS assessment was performed at the bedside in the ICU by measuring the IVC diameter and venous flow patterns of the hepatic, portal and intrarenal veins, with concurrent ECG tracing. The VExUS C grading system was used for score interpretation, following the criteria established by Beaubien-Souligny et al. [7].

### Sample size calculation

The sample size was originally calculated to estimate the proportion of patients demonstrating improvement in IRVF patterns following fluid removal. A literature review indicated that 60% of 15 patients showed improved IRVF patterns after two days of decongestive treatment [11]. Using a 95% confidence level and a margin of error of 0.15, the required sample size was calculated to be 41 patients. To account for potential data loss, a 20% buffer was applied, resulting in a final sample size of 52 patients. While this calculation was appropriate for the initial descriptive objective, the current study also investigates associations between IRVF patterns and fluid removal outcomes. As such, the study may be underpowered to detect these associations with high precision.

### Statistical analysis

Continuous variables were compared using the independent samples t test for normally distributed data and the Mann–Whitney U test for nonnormally distributed data. Categorical variables were analyzed using Pearson’s χ^2^ test or Fisher’s exact test, as appropriate. Probability (P) values < 0.05 were considered statistically significant.

To evaluate the diagnostic performance of baseline parameters in identifying successful fluid removal, sensitivity, specificity, and accuracy, along with corresponding 95% confidence intervals, were calculated.

To identify factors independently associated with the primary outcome, a two-step modeling approach was used. First, univariate logistic regression was performed for all clinically relevant baseline predictors to screen for potential associations. Second, all variables with a P value < 0.1 in the univariate analysis were then included in a multivariate logistic regression model using the "Enter" method to identify independent predictors of successful fluid removal.

To evaluate changes in secondary outcomes over time, appropriate methods for longitudinal data were used, including two-way repeated measures ANOVA, linear mixed-effects models, or the Mann–Whitney U test on change scores, after confirming that the assumptions for these analyses were met.

All analyses were performed using IBM SPSS Statistics for Windows, Version 29.0.2.0 (IBM Corp, Armonk, NY, USA).

## Results

**Fig. 3:**
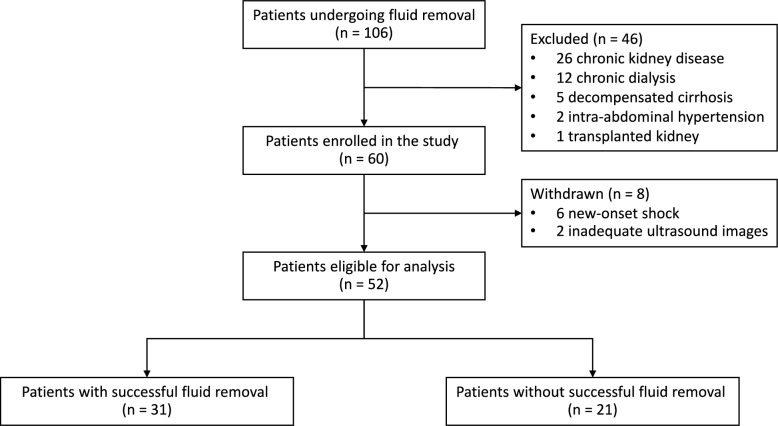
Study flow diagram

**Fig. 4:**
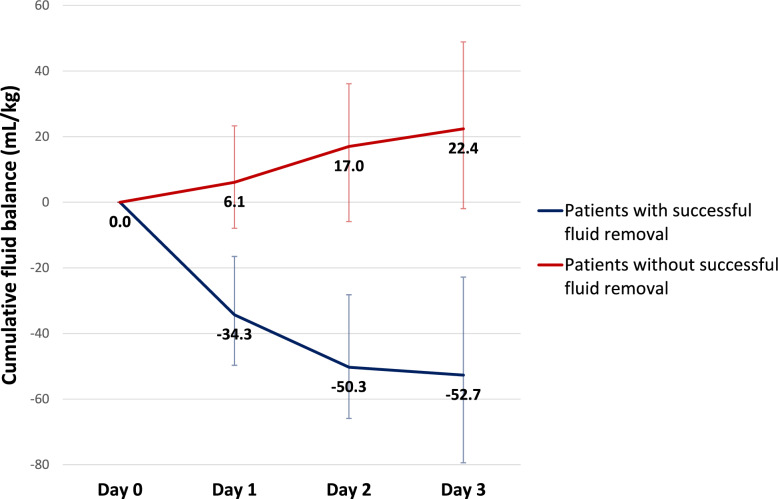
Median cumulative fluid balance over the first three days. The plot shows the median cumulative fluid balance (mL/kg) for each group: patients who achieved fluid removal (blue line) and those who did not (red line). Error bars represent the interquartile range (IQR). From Day 1 to Day 3 the median cumulative fluid balance was significantly more negative in the achieved group compared to the not achieved group, with a P value < 0.01 at every time point

A total of 106 patients were screened for eligibility, of whom 46 were excluded and 8 were subsequently withdrawn. In total, 52 patients were included in the analysis. Among them, 31 patients (59.6%) achieved successful fluid removal, while 21 patients (40.4%) did not (Fig. 3). Daily and cumulative fluid balances were significantly more negative in the achieved group across all three days (− 52.7 (-79.4, -22.8) vs. 22.4 (-1.95, 48.9) mL/kg, P < 0.01) (Fig. 4).

Baseline characteristics, including age, sex, disease severity, and most comorbidities, were comparable between the two groups. However, liver disease was more prevalent in the non-achieved group (0.0% vs 19.0%, P = 0.022). More than 70% of the study population presented with shock, with septic shock the most common (63.5%). The median maximum norepinephrine-equivalent dose before enrollment was 0.12 (0.09–0.21) µg/kg/min.

The cumulative fluid balance from admission to enrollment was similar between the groups (76.30 vs. 74.60 mL/kg, P = 0.948). At enrollment, mean arterial pressure (MAP) was also comparable (90 ± 14 vs. 90 ± 15 mmHg, P = 0.906). Although the CVP was slightly higher in the achieved group, the difference was not statistically significant (12 ± 3 vs. 10 ± 4 mmHg, P = 0.182).

The presence of pulmonary rales was significantly higher among patients with successful fluid removal compared to those without (87.1% vs. 57.1%, P = 0.014). Pulmonary edema on chest X-ray was more frequent and NT-proBNP levels were higher in patients with successful fluid removal, but these differences did not reach statistical significance (45.2% vs. 19.0%, P = 0.052 and 4510 vs. 3416 pg/mL, P = 0.145, respectively). The echocardiographic parameters were also comparable between the two groups (Table 1).

**Table 1:** Baseline characteristics

	All (n = 52)	Patients with successful fluid removal (n = 31)	Patients without successful fluid removal (n = 21)	P value
Characteristics				
Age, years (mean ± SD)	71 ± 14	72 ± 14	69 ± 15	0.407
Male, n (%)	26 (50.0)	18 (58.1)	8 (38.1)	0.158
Body weight at admission, kg, (mean ± SD)	60.67 ± 13.44	61.97 ± 14.81	58.74 ± 11.18	0.400
Body mass index, kg/m^2^ (mean ± SD)	23.34 ± 4.51	23.58 ± 4.71	22.98 ± 4.28	0.641
APACHE II score (mean ± SD)	20 ± 7	20 ± 7	21 ± 7	0.480
SOFA score (mean ± SD)	8 ± 3	9 ± 3	7 ± 4	0.182
Baseline serum creatinine, mg/dL, median (mean ± SD)	1.02 ± 0.46	1.12 ± 0.46	0.87 ± 0.42	0.052
Baseline eGFR, mL/min/1.73m2, median (mean ± SD)	70.14 ± 27.71	64.31 ± 26.90	78.75 ± 27.24	0.065
Source of ICU admission				
Emergency department, n (%)	29 (55.8)	18 (58.1)	11 (52.4)	0.686
In-hospital transfer, n (%)	23 (42.2)	13 (41.9)	10 (47.6)	0.686
Comorbidities				
Diabetes mellitus, n (%)	25 (48.1)	18 (58.1)	7 (33.3)	0.080
Hypertension, n (%)	32 (61.5)	21 (67.7)	11 (52.4)	0.264
Liver disease, n (%)	4 (7.7)	0 (0.0)	4 (19.0)	0.022
Cancer, n (%)	12 (23.1)	5 (16.1)	7 (33.3)	0.188
Coronary artery disease, n (%)	9 (17.3)	7 (22.6)	2 (9.5)	0.283
Immunosuppression, n (%)	6 (11.5)	3 (9.7)	3 (14.3)	0.675
Cerebrovascular disease, n (%)	18 (34.6)	14 (45.2)	4 (19.0)	0.052
Anemia (Hct < 30%), n (%)	8 (15.4)	5 (16.1)	3 (14.3)	1.000
Chronic kidney disease (eGFR > 30 mL/min/m2), n (%)	8 (15.4)	6 (19.4)	2 (9.5)	0.449
Shock in visit, n (%)	38 (73.1)	22 (71.0)	16 (76.2)	0.677
Septic shock, n (%)	33 (63.5)	19 (61.3)	14 (66.7)	0.693
Hypovolemic shock, n (%)	2 (3.8)	1 (3.2)	1 (4.8)	1.000
Cardiogenic shock, n (%)	4 (7.7)	3 (9.7)	1 (4.8)	0.639
Obstructive shock, n (%)	1 (1.9)	1 (3.2)	0 (0.0)	1.000
Vasopressor use, n (%)	36 (69.2)	21 (67.7)	15 (71.4)	0.777
Maximum Norepinephrine equivalent dose before enrollment, µg/kg/min, median (IQR) (n = 36)	0.12 (0.09, 0.21)	0.12 (0.09, 0.24)	0.13 (0.09, 0.21)	0.924
At enrollment				
Cumulative fluid balance from hospital admission, mL/kg, median (IQR)	75.45 (45.60, 139.10)	76.30 (44.60, 138.20)	74.60 (45.50, 143.30)	0.948
Cumulative fluid balance from ICU admission, mL/kg, median (IQR)	57.85 (37.40, 103.58)	56.30 (36.00, 96.90)	58.00 (42.20, 131.70)	0.412
MAP at enrollment, mmHg, (mean ± SD)	90 ± 14	90 ± 14	90 ± 15	0.906
CVP at enrollment, mmHg, (mean ± SD) (n = 36)	11 ± 4	12 ± 3	10 ± 4	0.182
Vasopressor at enrollment, n (%)	22 (42.3)	13 (41.9)	9 (42.9)	0.947
Norepinephrine equivalent dose at enrollment, µg/kg/min, median (IQR) (n = 22)	0.05 (0.03, 0.08)	0.04 (0.03, 0.08)	0.05 (0.02, 0.09)	0.948
Invasive mechanical ventilation, n (%)	46 (88.5)	28 (90.3)	18 (85.7)	0.675
Clinical manifestation at enrollment				
Pedal edema, n (%)	9 (17.3)	6 (19.4)	3 (14.3)	0.724
Pulmonary rales, n (%)	39 (75.0)	27 (87.1)	12 (57.1)	0.014
Chest X-ray findings				
Pulmonary edema, n (%)	18 (34.6)	14 (45.2)	4 (19.0)	0.052
Pleural effusion, n (%)	16 (30.8)	10 (32.3)	6 (28.6)	0.777
Baseline laboratory at enrollment				
NT-proBNP, pg/mL, median (IQR) (n = 39)	3657 (1994, 8711)	4510 (2425, 11,196)	3416 (1302, 7036)	0.145
Lactate, mmol/L, median (IQR)	1.7 (1.3, 2.2)	1.7 (1.2, 2.1)	1.6 (1.3, 2.3)	0.607
Echocardiographic parameters				
LVEF, %, mean ± SD (n = 51)	53 ± 19	51 ± 18	55 ± 19	0.460
E/A ratio, median (IQR) (n = 44)	1.14 (0.80, 1.74)	1.14 (0.78, 1.84)	1.13 (0.80, 1.58)	0.512
E/e’ratio, mean ± SD (n = 48)	16.28 ± 6.44	17.55 ± 6.38	14.16 ± 6.14	0.078
Moderate–severe TR, n (%)	3 (5.8)	2 (6.5)	1 (4.8)	1.000
RVSP, mmHg, median (IQR) (n = 41)	17.57 (12.62, 29.00)	17.57 (14.95, 31.65)	16.49 (13.09, 20.78)	0.227
TAPSE, mm, mean ± SD (n = 46)	18.97 ± 5.13	19.36 ± 4.38	18.37 ± 6.20	0.528
TAPSE/RVSP, mm/mmHg, median (IQR) (n = 40)	1.03 (0.59, 1.60)	1.02 (0.66, 1.42)	1.12 (0.50, 2.49)	0.525

APACHE II score, Acute Physiology and Chronic Health Evaluation II score; SOFA score, Sequential Organ Failure Assessment; eGFR, Estimated glomerular filtration rate; ICU, Intensive care unit; MAP, Mean arterial pressure; CVP, Central venous pressure; NT-proBNP, N-terminal prohormone of brain natriuretic peptide; LVEF, Left ventricular ejection fraction; E/A, Early to late diastolic transmitral flow velocity ratio; E/e’, Ratio of early diastolic transmitral flow to early diastolic mitral annular velocity; TR, Tricuspid regurgitation; RVSP, Right ventricular systolic pressure; TAPSE, Tricuspid annular plane systolic excursion

**Fig. 5:**
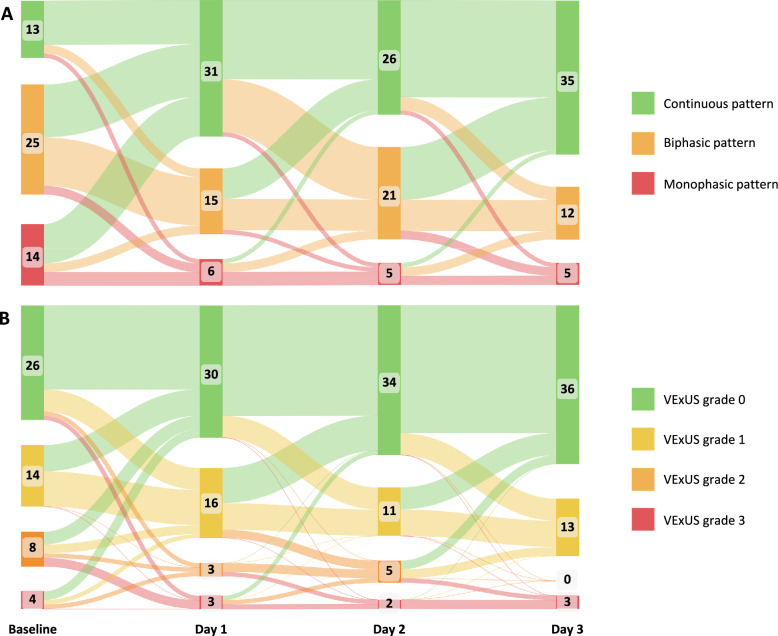
Temporal changes in intrarenal venous flow patterns and VExUS grades over the first three days. **A** Intrarenal venous flow patterns; **B** VExUS grades. VExUS, Venous Excess Ultrasound

At baseline, a discontinuous IRVF pattern was significantly more prevalent among patients who achieved successful fluid removal (87.1% vs. 57.1%, P = 0.014) (Table 2). The progression of the IRVF patterns and the VExUS scores over the three-day study period is illustrated in Fig. 5.

**Table 2:** Point-of-care ultrasonography assessments

	Patients with successful fluid removal (n = 31)	Patients without successful fluid removal (n = 21)	P value
IRVF pattern			
IRVF pattern: Baseline			
Continuous, n (%)	4 (12.9%)	9 (42.9%)	0.014
Discontinuous, n (%)	27 (87.1%)	12 (57.1%)	
IRVF pattern: Day 1			
Continuous, n (%)	19 (61.3%)	12 (57.1%)	0.765
Discontinuous, n (%)	12 (38.7%)	9 (42.9%)	
IRVF pattern: Day 2			
Continuous, n (%)	13 (41.9%)	13 (61.9%)	0.158
Discontinuous, n (%)	18 (58.1%)	8 (38.1%)	
IRVF pattern: Day 3			
Continuous, n (%)	20 (64.5%)	15 (71.4%)	0.602
Discontinuous, n (%)	11 (35.5%)	6 (28.6%)	
IVC: Baseline			
Maximal IVC diameter, mean ± SD	1.92 ± 0.48	1.92 ± 0.41	0.992
IVC collapsibility index (%), median (IQR)	15.50 (9.60, 26.00)	15.80 (8.30, 31.45)	0.615
IVC distensibility index (%), median (IQR)	18.30 (10.70, 35.20)	18.80 (9.05, 46.00)	0.608
Hepatic venous flow waveform pattern: Baseline			
Triphasic pattern, n (%)	15 (48.4%)	11 (52.4%)	0.585
S < D pattern, n (%)	8 (25.8%)	7 (33.3%)	
Reverse S pattern, n (%)	8 (25.8%)	3 (14.3%)	
Portal venous waveform: Baseline			
Portal venous pulsatility index (%), median (IQR)	33.00 (24.00,46.00)	33.00 (27.50, 45.00)	0.801
VExUS score: Baseline			
VExUS score, median (IQR)	1 (0,2)	0 (0,1)	0.572

IRVF, Intrarenal venous flow; IVC, Inferior vena cava; VExUS, Venous Excess Ultrasound

To identify independent predictors of the primary outcome, univariate and multivariate logistic regression analyses were performed. In the univariate analysis, a discontinuous IRVF pattern and a higher E/e' ratio were identified as potential predictors. When both variables were included in the multivariate model, only the discontinuous IRVF pattern at baseline remained a statistically significant independent predictor of successful fluid removal (adjusted odds ratio 4.31, 95% CI 1.02–18.18; P = 0.047) (Table 3).

**Table 3:** Univariate and multivariate logistic regression analysis for successful fluid removal

Baseline predictor	Univariate odds ratio (95%CI)	P value	Adjusted odds ratio (95%CI)	P value
VExUS components				
Discontinuous IRVF pattern	5.06 (1.30–19.72)	0.019	4.31 (1.02–18.18)	0.047
Abnormal hepatic venous flow pattern	1.17 (0.39–3.56)	0.778	-	-
Portal venous pulsatility index (per 1% increase)	0.99 (0.95–1.03)	0.628	-	-
Maximal IVC diameter (per 1 mm increase)	1.01 (0.29–3.53)	0.992	-	-
VExUS score (Grade 2–3 vs. 0–1)	2.46 (0.58–10.44)	0.224	-	-
Physiological and Echocardiographic Parameters				
CVP (per 1 mmHg increase)	1.14 (0.94–1.39)	0.184	-	-
E/A ratio (per 1-unit increase)	1.46 (0.63, 3.34)	0.376	-	-
E/e’ ratio (per 1-unit increase)	1.10 (0.99, 1.22)	0.084	1.08 (0.96–1.20)	0.192
TAPSE/RVSP ratio (per 1 mm/mmHg increase)	0.68 (0.36, 1.31)	0.250	-	-

VExUS, Venous Excess Ultrasound; IRVF, Intrarenal venous flow; IVC, Inferior vena cava; CVP, Central venous pressure; E/A, Early to late diastolic transmitral flow velocity ratio; E/e’, Ratio of early diastolic transmitral flow to early diastolic mitral annular velocity; TAPSE, Tricuspid annular plane systolic excursion; RVSP, Right ventricular systolic pressure

To characterize the diagnostic utility of the key ultrasound parameters, a discontinuous IRVF pattern demonstrated high sensitivity of 87.1% (95% CI 70.2–96.4), moderate specificity of 42.9% (95% CI 21.8–66.0), and overall accuracy of 69.2% (95% CI 54.9–81.3) for predicting successful fluid removal. In contrast, a baseline VExUS score grades 2–3 showed high specificity of 85.7% (95% CI 63.7–97.0) but low sensitivity of 29.0% (95% CI 14.2–48.0), with an overall accuracy of 51.9% (95% CI 37.6–66.0).

Other venous congestion assessment tools, including maximal IVC diameter, hepatic venous flow patterns, and portal venous pulsatility index, showed lower classification accuracies, ranging from 50.0% to 52.0%.

The daily doses of diuretics and utilization of renal replacement therapy did not differ significantly between the successful and unsuccessful fluid removal groups (Table 4). Improvement in the IRVF pattern from baseline to day 3 was significantly associated with a reduction in NT-proBNP levels (P = 0.048), but not with changes in cumulative fluid balance or CVP. Reduction in VExUS score was not significantly correlated with any of these parameters (Additional file 2). Furthermore, these changes were not associated with key clinical outcomes, including 28-day mortality, ventilator-free days, or ICU length of stay (Table 5).

**Table 4:** Details of fluid removal therapies by outcome group

Therapy details	Patients with successful fluid removal (n = 31)	Patients without successful fluid removal (n = 21)	P value
Day 1			
Patients on diuretics, n (%)	28 (90.3)	20 (95.2)	
Furosemide dose, mg, median (IQR)	40 (20, 160)	40 (13, 150)	0.784
Patients on RRT, n (%)	5 (16.1)	1 (4.8)	
UF Volume, mL, median (IQR)	2428 (566, 3940)	N/A*	N/A*
Day 2			
Patients on diuretics, n (%)	26 (83.9)	16 (76.2)	
Furosemide dose, mg, median (IQR)	50 (20, 100)	30 (10, 100)	0.333
Patients on RRT, n (%)	5 (16.1)	2 (9.5)	
UF Volume, mL, median (IQR)	2954 (1157, 3497)	N/A*	N/A*
Day 3			
Patients on diuretics, n (%)	21 (67.7)	13 (61.9)	
Furosemide dose, mg, median (IQR)	40 (20, 100)	40 (15, 530)	0.914
Patients on RRT, n (%)	4 (12.9)	1 (4.8)	
UF Volume, mL, median (IQR)	1081 (16, 2823)	N/A*	N/A*

RRT, Renal replacement therapy; UF, ultrafiltration

^*^Not calculated due to the very small sample size

**Table 5:** Clinical outcomes based on improvement in point-of-care ultrasonography parameters

	Improvement†	No improvement	P value
IRVF pattern	(n = 25)	(n = 14)	
28-day mortality, n (%)	6 (24.0)	2 (14.3)	0.686
ICU length of stay (days), median (IQR)	8.00 (5.00, 12.00)	10.00 (5.00, 19.25)	0.529
Hospital length of stay (days), median (IQR)	21.00 (13.00, 42.00)	27.50 (17.00, 42.25)	0.534
RRT-free days at day 28 after enrollment (days), median (IQR)	28.00 (10.00, 28.00)	28.00 (23.50, 28.00)	0.897
Ventilator-free days at day 28 after enrollment (days), median (IQR)	21.00 (0.50, 25.50)	24.00 (12.00, 24.25)	0.718
VExUS score	(n = 18)	(n = 8)	
28-day mortality, n (%)	4 (22.2)	1 (12.5)	1.000
ICU length of stay (days), median (IQR)	9.00 (5.00, 13.00)	7.50 (5.00, 13.25)	0.683
Hospital length of stay (days), median (IQR)	20.00 (13.00, 34.50)	22.50 (18.50, 37.75)	0.397
RRT-free days at day 28 after enrollment (days), median (IQR)	28.00 (21.00, 28.00)	28.00 (25.75, 28.00)	1.000
Ventilator-free days at day 28 after enrollment (days), median (IQR)	23.00 (13.50, 25.25)	23.50 (13.25, 24.75)	0.935

IRVF, Intrarenal venous flow; ICU, intensive care unit; RRT, Renal replacement therapy; VExUS, Venous excess ultrasound

^†^IRVF pattern improvement was defined as a transition from discontinuous to continuous pattern, and VExUS score improvement as a reduction in score from baseline to day 3

## Discussion

Among clinically stable ICU patients in the de-escalation phase of fluid management, the presence of a discontinuous IRVF pattern at baseline was significantly associated with successful fluid removal, demonstrating high sensitivity. This finding aligns with previous studies in heart failure populations, where discontinuous IRVF patterns were indicative of venous congestion and showed a transition to continuous patterns following effective decongestive therapy [9, 11]. Given that the kidneys are encapsulated organs, they are particularly susceptible to elevated venous pressure. Even mild venous congestion can lead to increased renal interstitial pressure and parenchymal compression, manifesting as discontinuous IRVF patterns​ [13]. However, the high VExUS scores were associated with better specificity but lower sensitivity, and did not significantly differ between groups at baseline. This may be explained by its systematic assessment of multiple sites of venous congestion, which strengthens its specificity [7, 14]. However, the 2 cm cut-off of the IVC diameter used in the initial step of the VExUS scoring system may reduce sensitivity, particularly in Asian populations [8].

An improvement in the IRVF pattern over three days was significantly associated with a reduction in NT-proBNP levels. This suggests a direct link between the relief of renal venous congestion and a reduction in myocardial wall stress. There are two potential physiological explanations for this. First, renal venous congestion is a key contributor to cardiorenal syndrome, where increased renal afterload can worsen cardiac function. Relieving this congestion may improve overall hemodynamics, thereby reducing the cardiac stretch that stimulates NT-proBNP secretion [15, 16]. Second, the kidneys play a role in the clearance of NT-proBNP. It is plausible that improving renal hemodynamics, as evidenced by the normalization of the IRVF pattern, enhances this clearance mechanism [17, 18]. In contrast, we did not find a significant correlation between changes in ultrasound parameters and changes in CVP or cumulative fluid balance. This may be because IRVF is a sensitive marker of tissue-level congestion, which may not correlate directly with systemic pressures or overall fluid balance shifts, especially in a heterogeneous ICU population [10, 12].

In this study, neither the IRVF pattern nor the VExUS score improvements were significantly associated with clinical outcomes such as 28-day mortality, ICU length of stay or ventilator-free days. Renal-related outcomes have been the most frequently examined clinical endpoints in previous studies. In patients with heart failure, discontinuous IRVF patterns have been associated with worsening renal function [9], and VExUS scores have shown a strong correlation with postoperative AKI [7]. However, in our study, there was no significant difference in RRT-free days, consistent with findings from other general ICU-based VExUS studies that have not demonstrated strong associations with renal outcomes [19–21]. In patients with severe AKI, a reduction in VExUS scores was associated with a higher number of RRT-free days [22]. Similarly, changes in IRVF patterns were not correlated with any clinical outcomes of interest [12]. This may be explained by the heterogeneous nature of the ICU population, where patients often present with multiple comorbidities and multiorgan dysfunction, making it difficult to demonstrate uniform clinical outcomes, as seen in populations with isolated cardiac disease.

Both IRVF and VExUS assessments proved feasible in this study, despite the fact that most patients were critically ill, mechanically ventilated, and diagnosed with septic shock. Similarly, previous studies demonstrated that, with appropriate training, bedside venous Doppler imaging can be reliably performed in the ICU, supporting its integration into routine practice [12, 21].

Owing to its high sensitivity to venous congestion and practical feasibility, the IRVF pattern appears to be a promising, physiology-based, non-invasive bedside tool to guide readiness for fluid removal in critically ill patients. In contrast, VExUS scores may serve as a confirmatory tool for systemic congestion and a monitoring strategy during the fluid removal process.

This study has several strengths. First, it is the prospective ICU study to assess serial IRVF patterns and VExUS scores from initiation and throughout the course of fluid removal, providing robust data on their role as fluid management tools. Second, blinding both attending physicians and ultrasound image interpreters minimized bias in clinical decision-making and outcome assessment. Third, the study focused on a diverse population of critically ill patients, including a high proportion with septic shock, improving the generalizability of the findings to real-world ICU settings. Finally, all ultrasound assessments were performed by a single trained operator using a standardized protocol, ensuring consistency and reliability of data collection.

However, this study has several limitations. The small sample size—originally calculated for a descriptive endpoint—reduces the statistical power and may limit the precision of the findings related to clinical outcomes. This was a single-center study, which limits its generalizability. A formal statistical analysis of intra-observer variability was not performed, although rigorous steps were taken to ensure measurement consistency. Additionally, fluid removal was not standardized, leading to physician-dependent variability in fluid management strategies. The discontinuation of de-escalation may have occurred due to poor tolerance or achievement of clinical goals. Finally, the lack of long-term follow-up beyond 28 days restricts the ability to evaluate long-term clinical implications.

Future research should focus on larger, multicenter studies to validate IRVF patterns and VExUS scores as reliable tools for fluid management. Standardizing fluid removal protocols may help reduce variability and improve comparability between studies. Additionally, integrating Doppler-based assessments with right ventricular-pulmonary arterial (RV-PA) coupling parameters and dynamic hemodynamic monitoring methods, such as passive leg raise testing or bioimpedance analysis, may enhance predictive accuracy. Extended follow-up periods will also be essential to better understand the association between markers of venous congestion and long-term clinical outcomes.

## Conclusion

In this prospective ICU study, discontinuous IRVF patterns were significantly associated with successful fluid removal, demonstrating high sensitivity as an early marker of venous congestion. The VExUS scores, while showing better specificity, may serve more effectively as confirmatory tools to support decisions about initiation of decongestive therapy. Although neither tool was clearly associated with clinical outcomes, both were feasible and informative in critically ill patients, including those with septic shock. These findings highlight the potential value of integrating physiological ultrasound parameters into fluid management strategies. Further large-scale studies are warranted to validate these results and investigate their impact on long-term clinical outcomes.

## Supplementary Information

Additional file 1.

Additional file 2.

## Data Availability

The datasets generated and/or analyzed during the study are available from the corresponding author upon reasonable request.
