# Supplementary material for: Intrarenal venous flow patterns and their association with successful fluid removal in critically ill patients: a prospective observational exploratory study
Source: Ultrasound J. 2025 Oct 6;17:44. doi: 10.1186/s13089-025-00447-z (PMC12501087; doi:10.1186/s13089-025-00447-z)
Supplement: Supplementary file 1 — Additional file 1. [file 13089_2025_447_MOESM1_ESM.docx]

**Additional File 1: Study Participants**

**Inclusion Criteria**

1. Adult patients 18 years or older admitted to the medical intensive care unit.
2. Expected to remain in the intensive care unit for more than 120 hours.
3. Stable vital signs without the need for vasoactive medications or rapid fluid resuscitation (defined as at least 500 milliliters of colloid or 1000 milliliters of crystalloid via rapid intravenous infusion) for a minimum consecutive period of 12 hours. This includes patients receiving norepinephrine at a rate not exceeding 0.1 micrograms per kilogram per minute and/or dobutamine at a rate not exceeding 10 micrograms per kilogram per minute, or those demonstrating a continuous reduction in vasoactive medications for a minimum of 12 consecutive hours.
4. Absence of signs suggestive of inadequate organ perfusion, such as mottled skin, a capillary refill time greater than 2 seconds following nailbed compression, or cold extremities.
5. Requirement for fluid removal, determined by attending staff or clinical guidelines, through diuretics or renal replacement therapy.

**Exclusion Criteria**

1. Chronic kidney disease with an estimated glomerular filtration rate (eGFR) < 30 mL/min/1.73m² prior to enrollment.
2. Chronic renal replacement therapy prior to enrollment (e.g., intermittent hemodialysis or peritoneal dialysis).
3. Decompensated cirrhosis with portal hypertension.
4. Thrombosis of the inferior vena cava, portal vein, hepatic veins, or renal veins.
5. Ureteral obstruction.
6. Intraabdominal hypertension (intraabdominal pressure > 12 mmHg).
7. Previous allergic or anaphylactic reactions to diuretics.
8. Pregnancy.
9. History of kidney or liver transplantation.
10. Patients with do-not-resuscitate (DNR) orders or decisions to withhold life-sustaining treatments.
11. Patients or their legally authorized representatives (LARs) who decline participation or are unable to provide informed consent before enrollment.

**Withdrawal or Termination Criteria**

1. New onset of shock requiring rapid fluid resuscitation (at least 500 milliliters of colloid or 1000 milliliters of crystalloid via rapid intravenous infusion) within 72 hours after enrollment.
2. Withdrawal from the study at the discretion of the patient, family, or attending physician.
