# Supplementary material for: Intrarenal venous flow patterns and their association with successful fluid removal in critically ill patients: a prospective observational exploratory study
Source: Ultrasound J. 2025 Oct 6;17:44. doi: 10.1186/s13089-025-00447-z (PMC12501087; doi:10.1186/s13089-025-00447-z)
Supplement: Supplementary file 2 — Additional file 2. [file 13089_2025_447_MOESM2_ESM.docx]

**Additional File 2: Correlations between improvements in IRVF and reductions in VExUS scores with CVP, NT-proBNP levels, cumulative fluid balance**

**
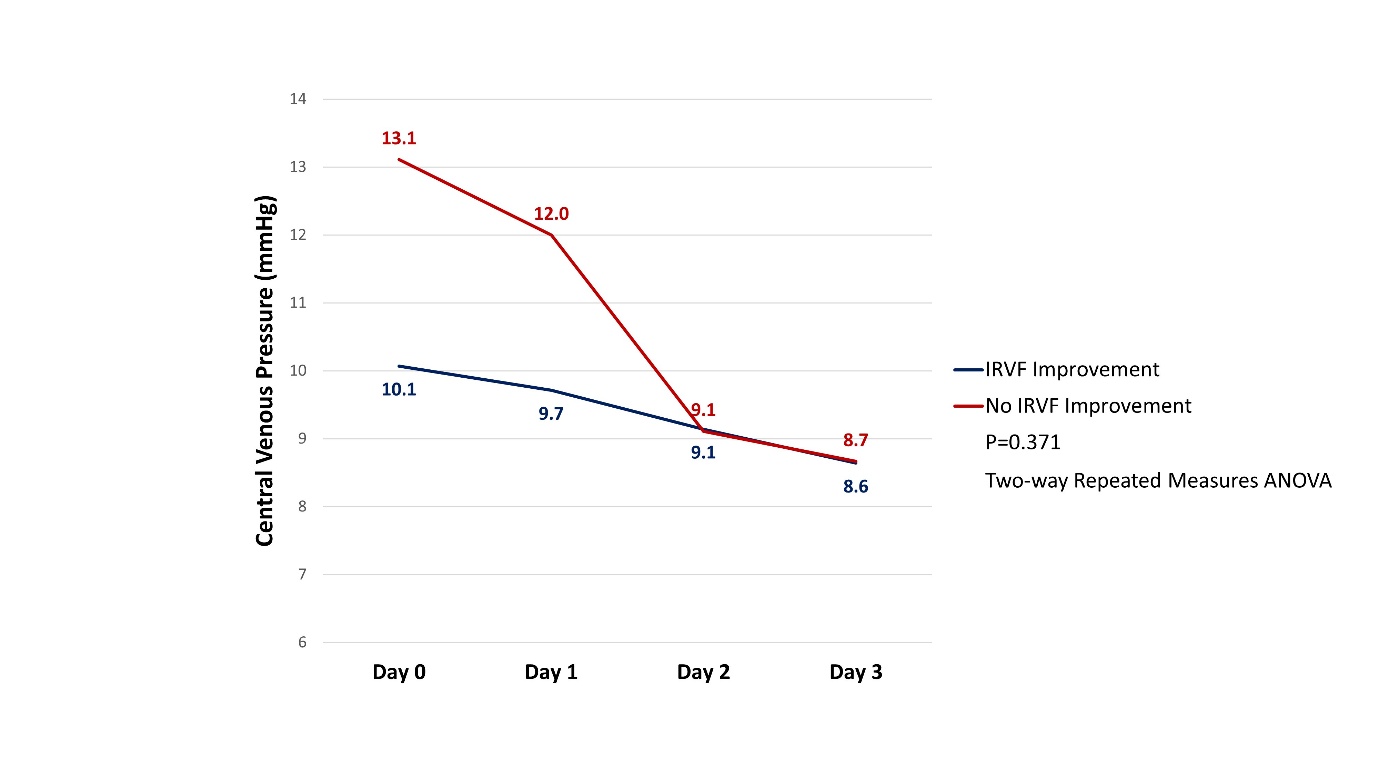
**


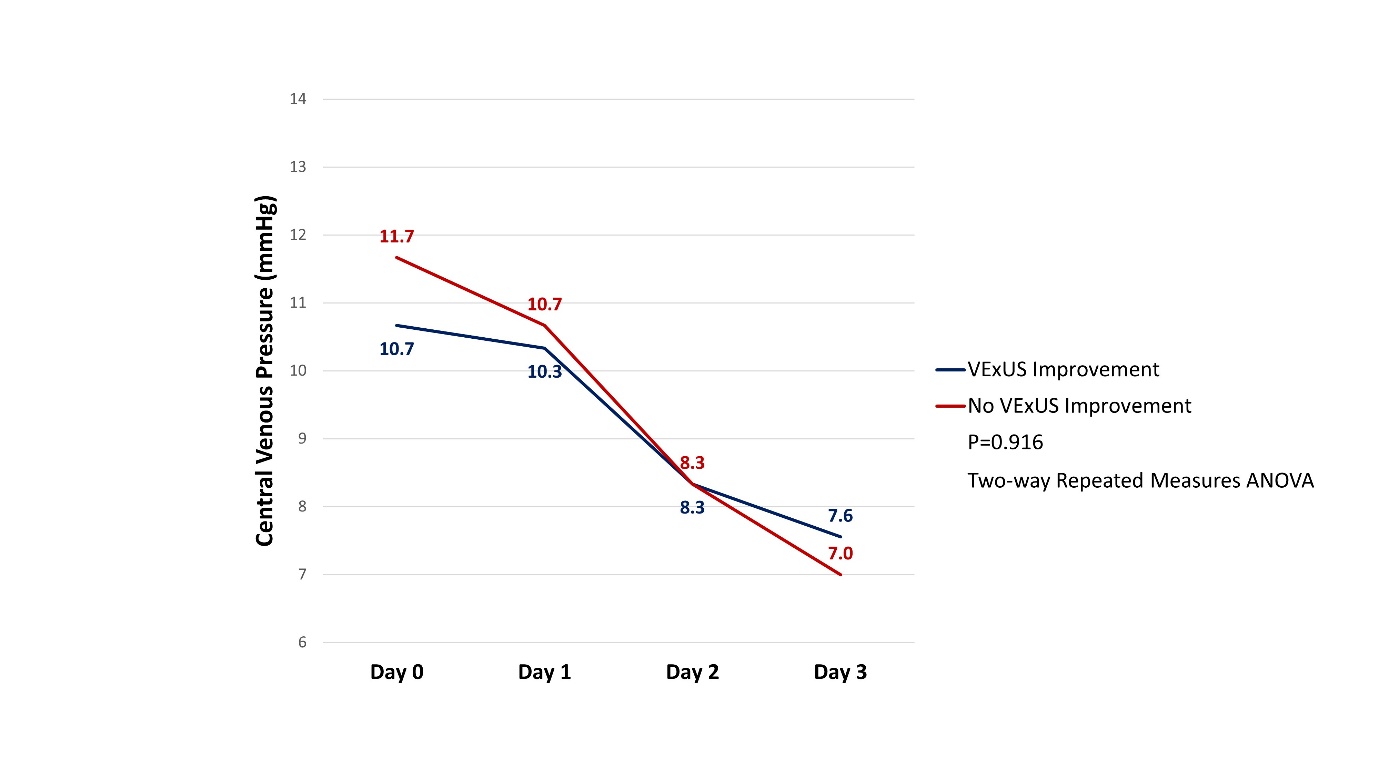


Correlations between improvements in IRVF and reductions in VExUS scores with CVP


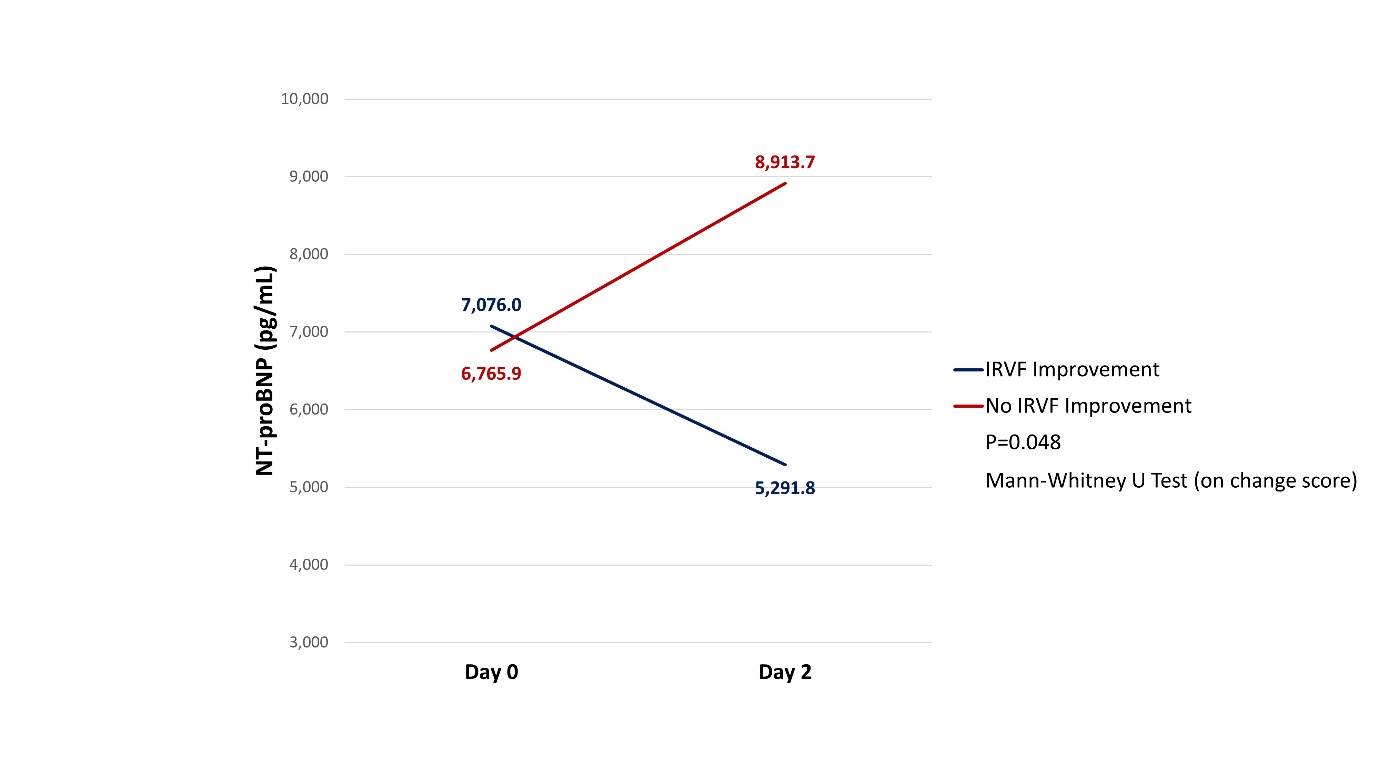

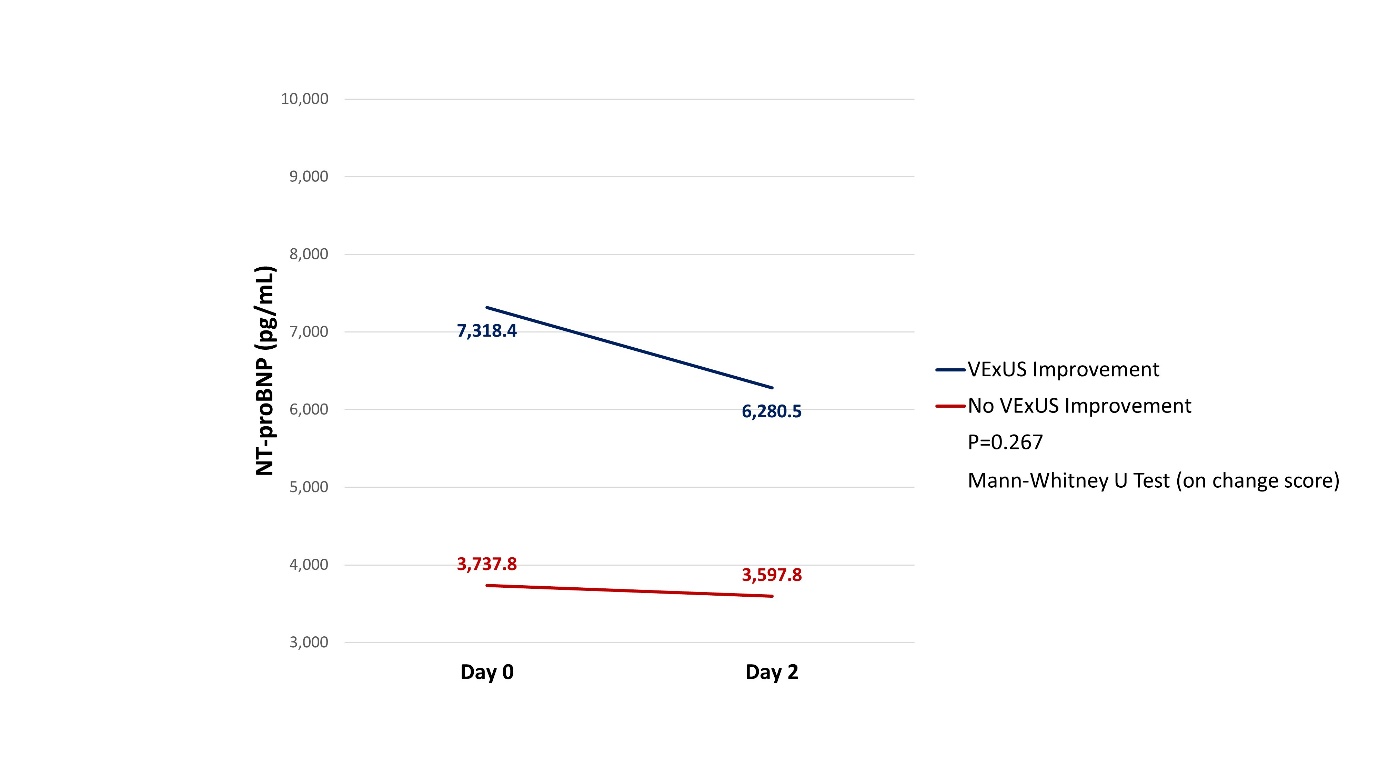


Correlations between improvements in IRVF and reductions in VExUS scores with NT-proBNP levels


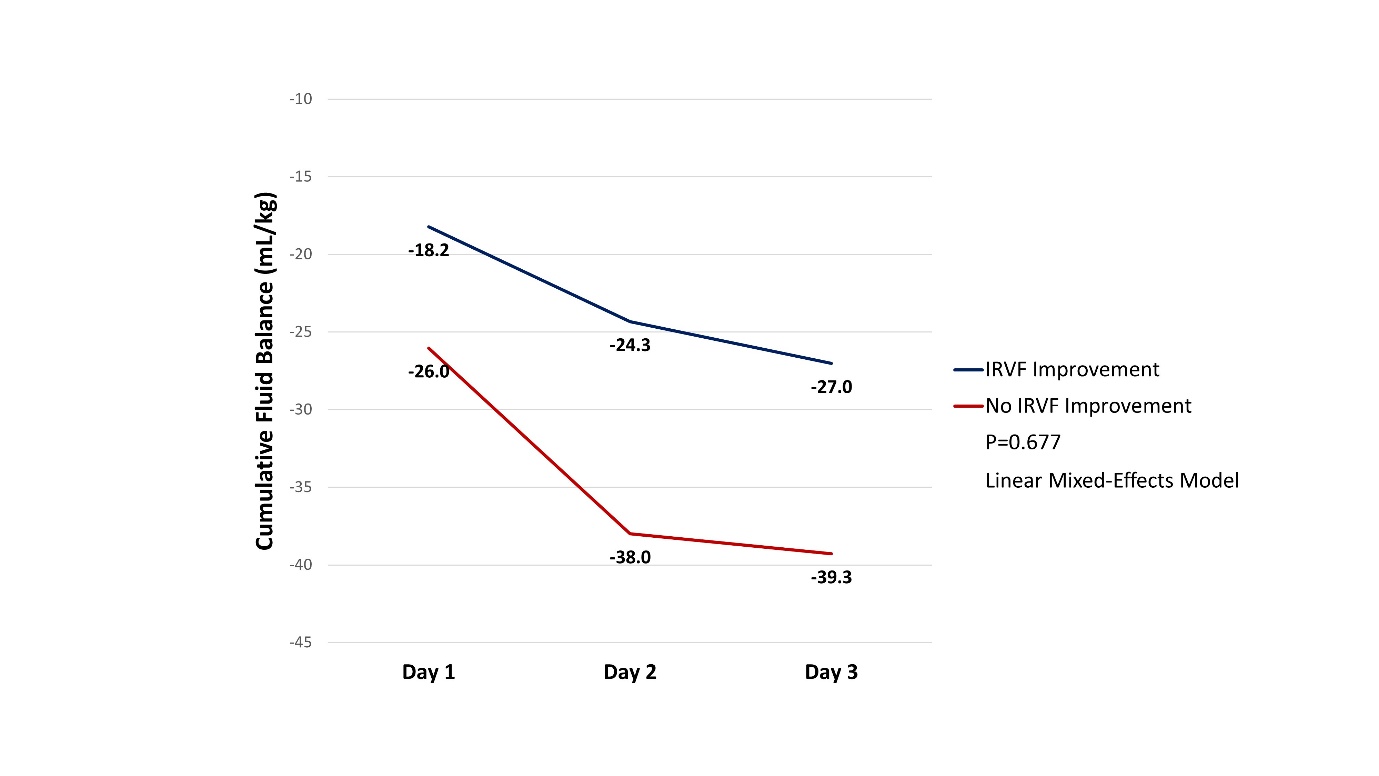

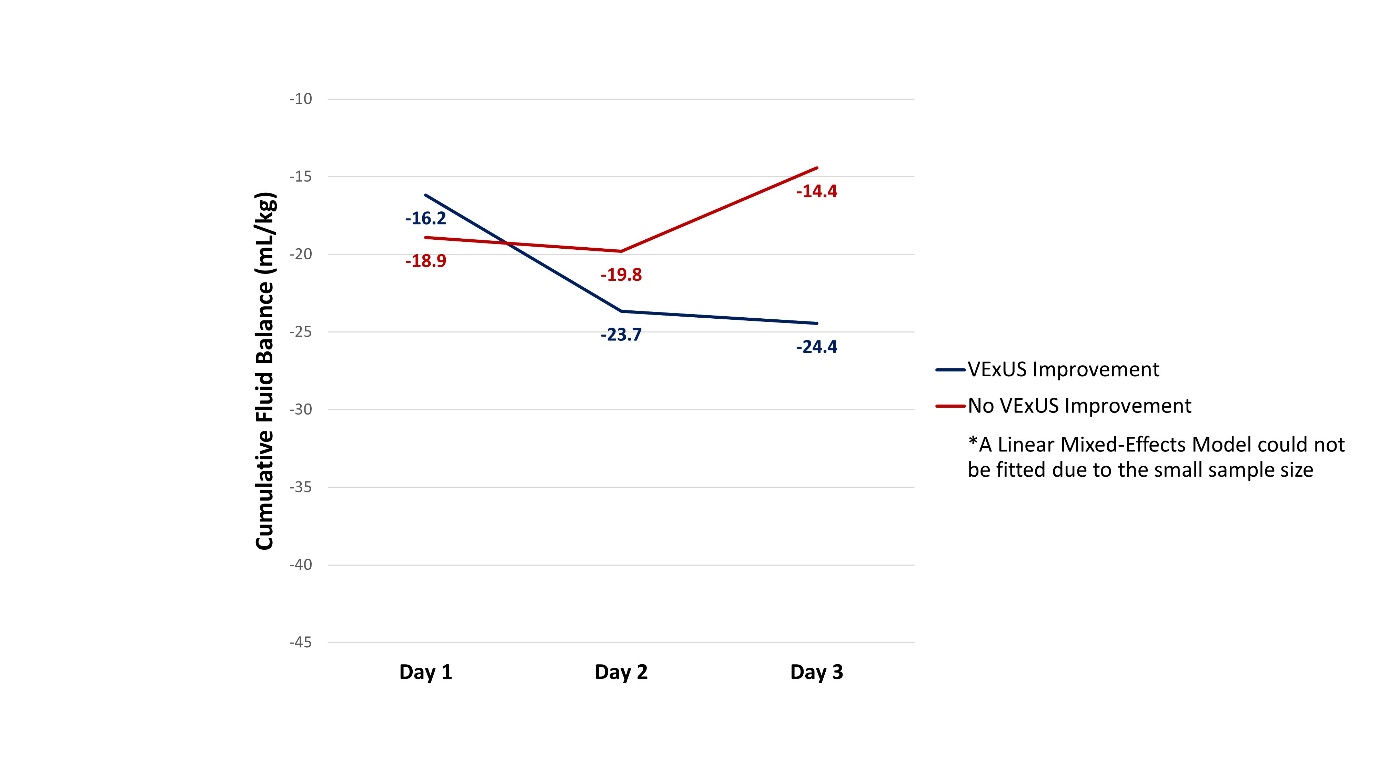


Correlations between improvements in IRVF and reductions in VExUS scores with cumulative fluid balance
